# Tumor protein 53‐induced nuclear protein 1 deficiency alters mouse gastrocnemius muscle function and bioenergetics in vivo

**DOI:** 10.14814/phy2.14055

**Published:** 2019-05-23

**Authors:** Julie Warnez‐Soulie, Michael Macia, Sophie Lac, Emilie Pecchi, Monique Bernard, David Bendahan, Marc Bartoli, Alice Carrier, Benoît Giannesini

**Affiliations:** ^1^ Aix Marseille Univ, INSERM, MMG Marseille France; ^2^ Aix Marseille Univ, CNRS, CRMBM Marseille France; ^3^ Aix Marseille Univ, CNRS, INSERM, Institut Paoli‐Calmettes, CRCM Marseille France

**Keywords:** Insulin resistance, mitochondrial function, multimodal NMR, obesity, oxidative stress

## Abstract

Tumor protein 53‐induced nuclear protein 1 (TP53INP1) deficiency leads to oxidative stress‐associated obesity and insulin resistance. Although skeletal muscle has a predominant role in the development of metabolic syndrome, the bioenergetics and functional consequences of TP53INP1 deficiency upon this tissue remain undocumented. To clarify this issue, gastrocnemius muscle mechanical performance, energy metabolism, and anatomy were investigated in TP53INP1‐deficient and wild‐type mice using a multidisciplinary approach implementing noninvasive multimodal‐NMR techniques. TP53INP1 deficiency increased body adiposity but did not affect cytosolic oxidative stress, lipid content, and mitochondrial pool and capacity in myocyte. During a fatiguing bout of exercise, the in vivo oxidative ATP synthesis capacity was dramatically reduced in TP53INP1‐deficient mice despite ADP level (the main in vivo stimulator of mitochondrial respiration) did not differ between both genotypes. Moreover, TP53INP1 deficiency did not alter fatigue resistance but paradoxically increased the contractile force, whereas there were no differences for muscle fiber‐type distribution and calcium homeostasis between both genotypes. In addition, muscle proton efflux was decreased in TP53INP1‐deficient mice, thereby indicating a reduced blood supply. In conclusion, TP53INP1 plays a role in muscle function and bioenergetics through oxidative capacity impairment possibly as the consequence of abnormal mitochondrial respiration regulation and/or defective blood supply.

## Introduction

Tumor protein 53‐induced nuclear protein 1 (TP53INP1) was identified in the late 1990s independently by three different laboratories (Saadi et al. 2015). The gene encoding TP53INP1 belongs to a gene family composed of two members, namely Tp53inp1 and Tp53inp2 (also known as DOR, for “diabetes‐ and obesity‐regulated”), which are highly conserved in mammals (Carrier et al. 1999; Baumgartner et al. 2007). TP53INP1 is considered as a key stress protein with antioxidant‐associated tumor‐suppressive function (Gommeaux et al. 2007; Cano et al. 2009; N'Guessan et al. 2011). Two major mechanisms by which TP53INP1 contributes to stress response have been unveiled. First, in the nucleus, TP53INP1 regulates the transcriptional activity of p53 and p73 by direct interaction, and mediates the antioxidant activity of p53 (Tomasini et al. 2002, 2005; Cano et al. 2009). Second, independently of p53, TP53INP1 is involved in autophagy and more particularly mitophagy (Seillier et al. 2012, 2015).

It has been evidenced in cultured cells and in vivo that TP53INP1 deficiency causes a chronic cellular oxidative stress characterized by an increase in reactive oxidative species (ROS) level and a decrease in antioxidant defenses (Gommeaux et al. 2007; N'Guessan et al. 2011). Restoration of TP53INP1 expression in TP53‐deficient cells is able to reduce ROS content toward normal level, demonstrating the primary role of TP53INP1 in the antioxidant activity of p53 (Cano et al. 2009). Besides, recent experiments have reported that the chronic oxidative stress observed in the absence of TP53INP1 results from the accumulation of defective mitochondria in association with impaired PINK1/PARKIN mitophagy and massive intracellular accretion of lipid droplets (Seillier et al. 2015), which is in accordance with other studies showing that mitochondrial function is impaired in various mouse models deficient for enzymes and transcription factors involved in antioxidant defenses (Ho et al. 2004; Liu et al. 2009). In addition, given that mice lacking TP53INP1 are prone to develop ROS‐driven obesity and insulin resistance, it has been proposed that TP53INP1 protects from metabolic syndrome through a mechanism involving prevention of oxidative stress through mitochondrial homeostasis regulation (Cano et al. 2009; N'Guessan et al. 2011; Seillier et al. 2015). Yet, it must be kept in mind that skeletal muscle has a predominant role in the development of metabolic syndrome because it is one of the major organs participating in the assimilation, storage, and utilization of glucose provided by food (Marette et al. 2014).

The primary objective of this study was to determine whether skeletal muscle is involved in the development of metabolic syndrome induced by TP53INP1 lacking. We hypothesized that TP53INP1 deficiency causes accumulation of ROS and defective mitochondria in myofiber. Moreover, because excessive cytosolic oxidative stress as well as reduced oxidative capacity are usually associated with impairment of muscle function (Reid et al. 1994; Supinski et al. 1997; Moopanar and Allen 2005; Zolfaghari et al. 2015), our second objective was to determine whether lack of TP53INP1 impairs oxidative ATP flux and mechanical performance in exercising muscle.

To address these objectives, we have investigated the impact of TP53INP1 deficiency upon gastrocnemius muscle using a multidisciplinary approach combining in vivo and in vitro experiments in mutant mice generated in the laboratory in which TP53INP1 has been genetically inactivated (Gommeaux et al. 2007). Especially, we have assessed muscle function and bioenergetics noninvasively using magnetic resonance (MR) imaging and 31‐phosphorus (^31^P) MR spectroscopy. ^31^P‐MR spectroscopy is a unique and valuable technique for the dynamic and simultaneous monitoring of the acidosis and the levels of high‐energy phosphorylated compounds involved in muscle bioenergetics, thereby assessing a number of key variables depicting muscle oxidative capacity, ATP turnover and regulation in intact muscle (Kemp et al. 1995, 2015; Lanza et al. 2011; Prompers et al. 2014).

## Methods

### Animal model, care, and feeding

Twenty‐one 5‐month‐old male Tp53inp1^+/+^ (*n *=* *10) and Tp53inp1^−/−^ (*n *=* *11) mice generated from heterozygous (Tp53inp1^+/−^) pairs (Laboratoire d'Exploration Fonctionnelle de Luminy, Marseille, France) were used for these experiments. The generation of Tp53inp1^−/−^ mice (in which the gene *Trp53inp1* encoding murine TP53INP1 was inactivated by homologous recombination) on the C57BL/6 parental genetic background and their genotyping by PCR were described previously (Gommeaux et al. 2007; N'Guessan et al. 2011). All animal procedures were performed with the approval of the animal experiment committee of Aix Marseille University (permit number #07‐25012013) and under the supervision of BG (license to perform experiments #13.164 2008/11/25) in strict accordance with the guidelines of the European Communities Council Directive 86/609/EEC for Care and Use of Laboratory Animals. Every attempt was made to minimize the number and suffering of animals at all times. Mice were housed as 4–6 per cage in an environmentally controlled facility (12–12 h light–dark cycle, 22°C) with free access to commercial standard food and water until the time of the experiments.

### Study design

Each animal was studied in a noninvasive manner twice over a 1‐week period. The first study session was devoted to the dynamic in vivo investigation of gastrocnemius muscle function and bioenergetics throughout a standardized rest‐exercise‐recovery protocol including a fatiguing workout. During the second session, whole‐body MR images were acquired to quantify body and abdominal fat contents. Afterward, anesthetized animals were euthanatized by cervical dislocation. Gastrocnemius muscles were quickly removed, freeze‐clamped with nitrogen‐chilled metal tongs, and stored at −80°C for in vitro analytical procedures.

### Noninvasive investigation of gastrocnemius muscle function and bioenergetics

Investigations were conducted using an innovative homebuilt experimental setup that has been designed to be operational inside the 4.7‐Tesla horizontal magnet of a preclinical 47/30 Biospec Avance MR scanner (Bruker, Karlsruhe, Germany) equipped with a 120‐mm BGA12SL (200 mT/m) gradient insert (Giannesini et al. 2010). The setup allows (1) to get anatomical information about the hind limb using MR imaging, (2) to assess gastrocnemius muscle mechanical performance with a dedicated ergometer consisting of a foot pedal coupled to a force transducer, and (3) to monitor dynamically the levels of high‐energy phosphorylated compounds and acidosis in this muscle using ^31^P‐MR spectroscopy. We have chosen to study the gastrocnemius muscle, which forms the belly of the calf, because it is clearly distinct from the other muscles of the leg, easily accessible for MR coils and large enough to give ^31^P‐MR spectra in a short time and with a good signal‐to‐noise ratio, and preferentially activated using our experimental methodology.

#### Animal preparation

Each mouse was initially anesthetized in an induction chamber using an air flow (3 L/min) containing 4% isoflurane. The left hind limb was shaved before electrode cream was applied at the knee and heel regions to optimize electrically evoked muscle contractions. Anesthetized animal was then placed supine in the experimental setup. Corneas were protected from drying by applying ophthalmic cream, and the animal's head was placed in a facemask continuously supplied with 1.75% isoflurane in 33% O_2_ (0.2 L/min) and 66% N_2_O (0.4 L/min). The foot was positioned on the pedal of the ergometer and the hind limb was centered inside a 20‐mm‐diameter ^1^H Helmholtz imaging coil while the belly of the gastrocnemius muscle was located above an elliptic (8 × 12 mm^2^) ^31^P‐MR spectroscopy surface coil. The leg was immobilized using piece works fitting animal morphology. Body temperature was controlled and maintained at a physiological level throughout the experiment using a feedback loop including an electrical heating blanket, a temperature control unit (ref. 507137; Harvard Apparatus, Holliston, MA, USA) and a rectal thermometer.

#### Induction of muscle contraction and contractile force measurement

Muscle contractions were induced by electrostimulation using two transcutaneous surface electrodes connected to a constant‐current stimulator (DS7A, Digitimer, Herthfordshire, UK). One electrode was placed at the heel level and the other one was located just above the knee joint. Electrical signal coming out from the force transducer of the ergometer was amplified (operational amplifier AD620; Analog Devices, Norwood, MA, USA; 70‐dB gain; 0–5 kHz bandwidth) and converted to a digital signal (PCI‐6220; National Instrument, Austin, TX, USA) that was continuously monitored and recorded on a personal computer using the WinATS software version 6.5 (Sysma, Aix‐en‐Provence, France). The digital signal was converted to force according to a linear calibration curve and expressed in mN.

#### Fatiguing exercise protocol

Gastrocnemius muscle function and bioenergetics were evaluated throughout a fatiguing bout of exercise consisting of 6 min of maximal isometric contractions repeated at a frequency of 1.7 Hz in order to produce marked mechanical and metabolic changes.

#### Preliminary adjustments

Before the launch of MR acquisition, muscle was passively stretched at rest by adjusting the angle between the foot and the hind limb in order to produce maximal twitch tension in response to supramaximal square wave pulses (1‐ms duration). The individual maximal electrostimulation intensity was determined by progressively increasing stimulus intensity until there was no further increase in the peak twitch force.

#### Multimodal MR data acquisition

Ten consecutive noncontiguous axial slices (1‐mm thickness; 0.5‐mm spaced) covering the region from the knee to the ankle were selected across the hind limb. Anatomic images of these slices were acquired at rest using a spin echo sequence (18.2‐ms echo time; 1000‐ms repetition time; two accumulations; 30 × 30 mm^2^ field of view; 256 × 256 matrix size; 8.5‐min total acquisition time). ^31^P‐MR spectra (8 kHz sweep width; 2048 data points) from the gastrocnemius muscle were continuously acquired before (rest period; 6‐min duration), during and after (recovery period; 15‐min duration) the 6‐min fatiguing exercise. Spectra acquisition was gated to muscle electrostimulation to reduce potential motion artifacts due to contraction. A fully relaxed spectrum (12 scans, 20‐sec repetition time) was acquired at rest, followed by a total of 768 saturated free induction decays (FID; 1.875‐sec repetition time). The first 64 FIDs were acquired in resting muscle and summed together. The next 192 FIDs were acquired during the exercise and were summed by packets of 32, allowing a 60‐sec temporal resolution. The remaining 512 FIDs were obtained during the recovery period and were summed as seven packets of 32 FIDs followed by three packets of 64 FIDs and one packet of 96 FIDs.

#### MR data processing and calculation

MR data were processed using custom‐written analysis programs developed under the Interactive Data Language (Exelis Visual Information Solutions, Boulder, CO, USA) environment (Mattei et al. 2006; Le Fur et al. 2010). For each hind limb MR image, region of interest was manually outlined so that the corresponding cross‐sectional area of the gastrocnemius muscle was measured. Muscle volume was calculated by summing up the volume between consecutive slices. Relative concentrations of phosphocreatine (PCr), inorganic phosphate (P_i_) and ATP were obtained from ^31^P‐MR spectra using a time domain fitting routine based on the AMARES‐MRUI Fortran code and appropriate prior knowledge for the ATP multiplets (Vanhamme et al. 1997). Absolute amounts of phosphorylated compounds were expressed relative to basal ATP concentration determined in vitro using a bioluminescence‐based method (see analytical procedures below). Intracellular pH was calculated from the chemical shift of the P_i_ signal relative to PCr according to the formula (Arnold et al. 1984): pH = 6.75 + log[(3.27 − *δ*_Pi_)/(*δ*_Pi_ − 5.69)]. ADP concentration was calculated from [PCr], [ATP] and pH considering the equilibrium constant (*K *=* *1.67 10^9^/M) of the creatine kinase reaction (Roth and Weiner 1991). The rate of PCr degradation (*V*PCr_stim_, in mM/min) at the start of the exercise was calculated as *V*PCr_stim_ = ΔPCr/*τ*PCr_stim_, where ΔPCr is the extent of PCr depletion measured at the exercise end (relative to basal value) and *τ*PCr_stim_ is the time constant of PCr degradation. *τ*PCr_stim_ was determined by fitting the time course of PCr level to a mono‐exponential function with a least‐mean‐squared algorithm. Similarly, the PCr recovery kinetic parameters were determined during the postexercise period by fitting the time course of PCr resynthesis to a mono‐exponential function. The maximal oxidative capacity (*Q*_max_, in mM/min) was calculated from the time constant of PCr resynthesis (*τ*PCr_rec_) and the PCr concentration in resting muscle (PCr_rest_): *Q*_max_ = PCr_rest_/*τ*PCr_rec_ (Lanza et al. 2011; Prompers et al. 2014). Force‐normalized oxidative ATP synthesis was calculated as the ratio between *V*PCr_rec_ and the amount of force produced at the exercise end considering that at this time, ATP production from oxidative phosphorylation is equal to *V*PCr_rec_ (Boska 1991; Prompers et al. 2014). The rate of proton efflux from the muscle (in mM/min) was calculated in the early stage of the postexercise recovery period from the changes in pH and levels of high‐energy phosphorylated contents as described previously (Trenell et al. 2006; Layec et al. 2011).

### Noninvasive MR measurement of fat content

Explorations were performed with our preclinical 47/30 Biospec Avance MR scanner. Each mouse was initially anesthetized by isoflurane inhalation as described above and placed in prone position in a whole‐body imaging coil (PRK200‐RES200, Bruker, Karlsruhe, Germany) integrating a facemask continuously supplied with 1.75% isoflurane in 33% O_2_ (0.2 L/min) and 66% N_2_O (0.4 L/min). Axial MR images were acquired across the entire body length excluding the tail using a turbo spin echo sequence (5.530‐ms echo time; 77.85 ms‐effective echo time; 300‐ms repetition time; 2 averages; 40 × 40 × 80 mm^3^ field of view, 128 × 128 × 64 matrix size). Fat volume was quantified using an automatic segmentation method based on a pixel intensity analysis of MR images with the FMRIB Software Library v5.0.2.2, (FSL, Oxford University, UK; http://www.fmrib.ox.ac.uk/fsl). Fat mass was calculated considering that density of adipose tissue is 0.92 g/cm^3^ (Farvid et al. 2005).

### Citrate synthase activity and metabolic content

Freeze‐clamped gastrocnemius muscle (20–30 mg) was homogenized with a lysis reagent (ref. C3228; Sigma‐Aldrich France, Saint‐Quentin Fallavier, France) and a protease inhibitor cocktail (ref. P834; Sigma‐Aldrich France). Citrate synthase activity was measured on a microplate reader (Victor X3; PerkinElmer, Waltham, MA, USA) using the colorimetric Citrate Synthase Assay Kit (ref. CS0720; Sigma‐Aldrich France) according to the manufacturer's instructions. Citrate synthase activity was normalized by the protein content measured using the colorimetric Pierce BCA Protein Assay Kit (ref. 23225; Thermo Fisher Scientific, Waltham, MA, USA).

Intramuscular contents for ATP, glycogen and glucose were determined in 40–60 mg of freeze‐clamped muscles homogenized in 1.2 mL of ice‐cold 0.6 M perchloric acid using a Polytron PT2100 (Kinematica AG, Luzern, Switzerland). After incubation for 15 min at 4°C, the homogenates were centrifuged (15 min, 2000 × *g*, 4°C). The supernatants were neutralized with K_2_CO_3_, placed for 30 min at 4°C and centrifuged (15 min, 2000 × *g*, 4°C) to remove precipitates. ATP concentration was determined using the bioluminescence ATP Determination Kit (ref. A22066; Invitrogen, Eugene, OR, USA). Glycogen and glucose contents were assessed by colorimetric procedure using the Glycogen Assay Kit (ref. E2GN‐100; EnzyChrome, Hayward, CA, USA).

Intramyocellular lipid (IMCL) content was determined in 50–70 mg of the freeze‐clamped muscle homogenized in 1 mL of a 1% (w/v) Triton X‐100 in chloroform solution. Briefly, homogenates were centrifuged (10 min, 13,000 × *g*, 20°C), the organic phases were collected and chloroform was removed using a nitrogen evaporator (N‐EVAP‐111, Organomation, Berlin, MA, USA). IMCL content was then measured using the colorimetric Fatty Acid Quantitation Kit (ref. MAK044, Sigma‐Aldrich France).

### Immunoblotting

#### Myosin heavy chain isoform, STIM1, and oxidatively modified proteins

Freeze‐clamped muscles were homogenized using a MagNA Lyser Instrument (Roche Life Science France, Meylan, France) in MagNA Lyser Green Beads filled with a lysis buffer containing 50 mM Tris/HCl, 15% glycerol, 1 mM DTT, 10 mM EDTA, 0.2% Triton, 46 mM KCl, and 10 μL of protease inhibitor cocktail (ref. P2714; Sigma‐Aldrich France) per 500 μL. The homogenates were subjected to sonication and centrifugation at 10,000 × *g* for 10 min at 4°C. The supernatants were aliquoted and used for BCA protein assay (Promega Glomax Multi detection System spectrophotometer at 560 nm) to determine total protein content. Twenty milligram of proteins extract was separated by SDS/PAGE and transferred onto nitrocellulose membranes (GE Healthcare). Membranes were probed with the appropriate primary antibodies for overnight at 4°C followed by washes and 1‐hour incubation at room temperature with the appropriate fluorescent secondary antibodies. For myosin heavy chain (MHC) isoform analysis, the primary antibodies were purchased from Developmental Studies Hybridoma Bank (University of Iowa, Iowa City, IA, USA) and were specific to the MHC type I (mouse anti‐bovine IgG2b, ref. BA‐F8; at 1:250), type IIa (mouse anti‐bovine IgG1, ref. SC‐71; at 1:250) and type IIb (mouse anti‐bovine IgGM, ref. BF‐F3; at 1:250). The primary antibody specific to glyceraldehydes 3‐phosphate dehydrogenase (GAPDH) was purchased from Santa Cruz Biotechnology (ref. SC‐48167; at 1:1000). The fluorescent secondary antibodies used were as follows: IRDye^®^ 800CW donkey anti‐mouse IgG (H + L) (ref. P/N926‐32212, Li‐Cor; at 1:10,000) and IRDye^®^ 800CW donkey anti‐goat IgG (H + L) (ref. P/N926‐32214, Li‐Cor; at 1:10,000). For STIM analysis, the specific primary antibody was purchased from Sigma‐Aldrich France (ref. S‐6072; at 1:750), and the fluorescent secondary antibodies used were as followed: IRDye^®^ 800CW donkey anti‐rabbit IgG (H + L) (ref. 926‐32213, Li‐Cor; at 1:10,000). For the oxidatively modified proteins analysis, specific primary antibody from the Oxidized Protein Western Blot Detection Kit (ref. ab178020; Abcam, Cambridge, UK), and fluorescent secondary antibody (ref. 926‐32213, Li‐Cor; at 1:10,000) were used. Proteins were detected using an Odyssey infrared imaging system (Odyssey, Li‐Cor Biosciences). Amplitudes of protein expression were arbitrary quantified with the ImageJ software (http://imagej.nih.gov/ij/). Peak area for each band corresponding to MYHx or STIM1 was normalized to GAPDH peak area.

#### PARKIN, PINK1, PGC‐1α, VDAC1, 4HNE analysis

Twenty micrometer sections from freeze‐clamped muscle were homogenized using polytron PT2100 in a lysis buffer containing 50 mM Tris/HCl, 15% glycerol, 1 mM DTT, 10 mM EDTA, 0.2% Triton, 46 mM KCl, and 10 μL of protease inhibitor cocktail (ref. P2714, Sigma‐Aldrich France) per 500 μL. The homogenates were subjected to sonication and centrifugation at 10,000 × *g* for 10 min at 4°C. The supernatants were aliquoted and used for BCA protein assay (Promega Glomax Multi detection System spectrophotometer at 560 nm) to determine total protein content. Twenty milligram of proteins extract was separated by SDS/PAGE and transferred onto nitrocellulose membranes (GE Healthcare). Membranes were blocked in 5% nonfat milk in phosphate‐buffered saline Tween‐20, and developed with antibodies specific for PARKIN (ab15954 – 1:1000, Abcam), PGC‐1*α* (ref. sc13067 – 1:200; Santa Cruz Biotechnology, Heidelberg, Germany), PINK1 (ref. BC100‐494 – 1:200, Novus Biologicals, Montluçon, France), VDAC1 (ref. ab14734 – 1:2500; Abcam, Cambridge, UK), 4HNE (ref. NHE11‐S – 1:500; Alpha Diagnostic, San Antonio, TX, USA), and *β*‐tubulin (ref. T4026 – 1:3000, Sigma‐Aldrich France). Secondary antibodies were purchased from Santa Cruz Biotechnology: anti‐rabbit horseradish peroxidase (HRP)‐conjugate (ref. sc‐2004 – 1:5000) and anti‐mouse HRP‐conjugate (ref. sc‐2005 – 1:5000). Immunoblots were developed using the Immobilon Western Chemiluminescent HRP Substrate (Millipore). Chemiluminescence was detected using Fusion FX7 device (Fisher Bioblock Scientific, Illkirch, France). Amplitudes of protein expression were arbitrary quantified with the ImageJ software (http://imagej.nih.gov/ij/). Peak area for each band corresponding to PARKIN, PINK1, PGC‐1*α*, VDAC1 or 4HNE was normalized to Beta‐tubulin peak area.

### Statistics

All values are expressed as mean ± SEM. Sample distribution was tested with the Shapiro–Wilk test. Force production and fatigue resistance throughout the 6‐min exercise were tested using two‐factor (group × time) analyses of variance with repeated measures on time followed by Tukey–Kramer post‐hoc multiple comparison tests to determine pairwise differences between groups. For other variables, significant differences were determined with nonparametric Mann–Whitney tests or parametric two‐tailed Student's *t*‐tests. The significance level was set at *P *<* *0.05.

## Results

### Morphological characteristics

**Figure 1:**
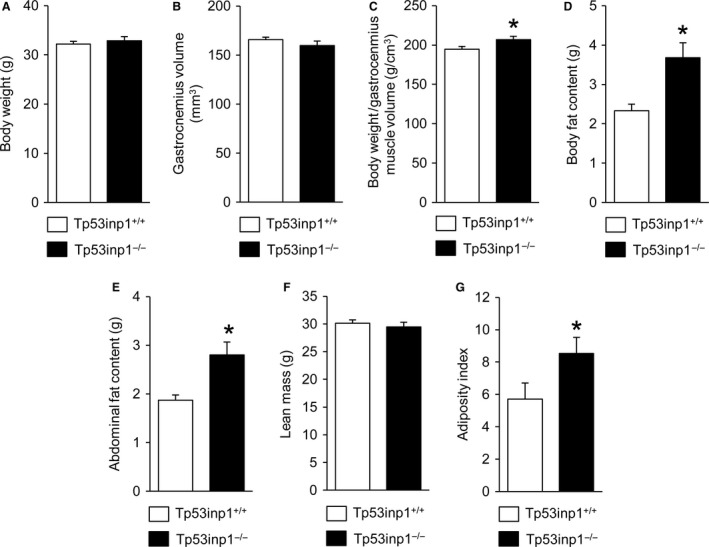
Morphological characteristics. Body weight (A), gastrocnemius muscle volume (B), ratio between body weight and gastrocnemius muscle volume (C), body (D) and abdominal (E) fat contents, lean mass (F), and adiposity index (G). Data are means ± SEM. *Significantly different from Tp53inp1^+/+^.

**Figure 2:**
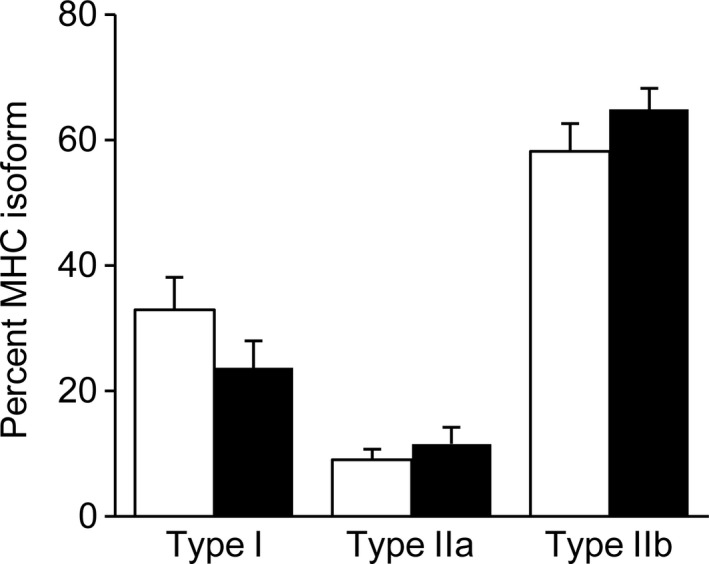
Gastrocnemius muscle typology. Relative distribution of myosin heavy chain (MHC) isoform proteins.

Body weight (Fig. 1A) and gastrocnemius muscle volume (Fig. 1B) did not differ significantly between wild‐type and TP53INP1‐deficient mice, but the ratio between body weight and gastrocnemius muscle volume was higher (+6%) in animals lacking TP53INP1 (Fig. 1C), hence suggesting that TP53INP1 deficiency affects body composition. We found using in vivo whole‐body MR imaging that both body (Fig. 1D) and abdominal (Fig. 1E) fat contents were larger (+56% and +50%, respectively) in TP53INP1‐deficient mice, whereas lean mass was not affected in those animals (Fig. 1F). Overall, adiposity index was 49% higher in mice lacking TP53INP1 (Fig. 1G). Besides, TP53INP1 deficiency did not alter the relative distribution of MHC isoform proteins in the gastrocnemius muscle (Fig. 2).

### Metabolic content

**Figure 3:**
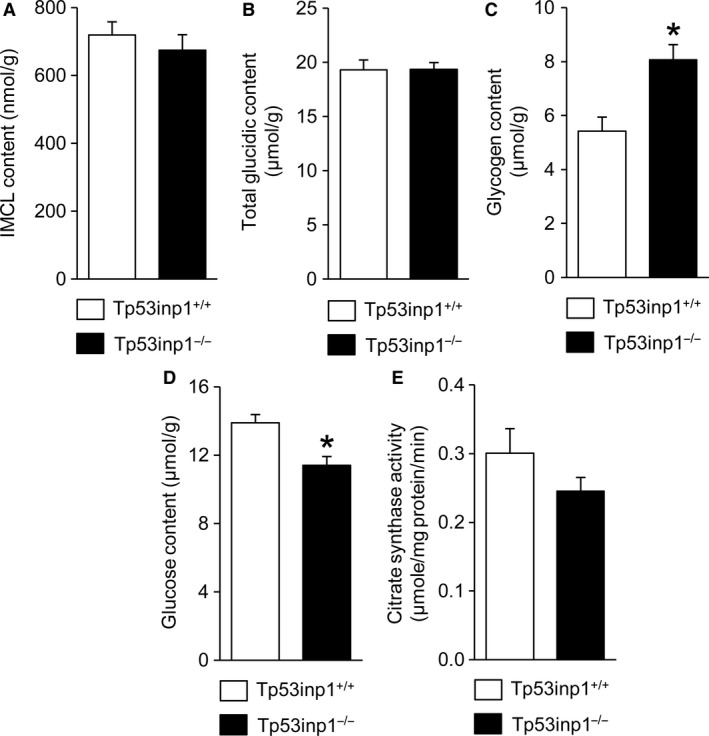
In vitro determination of metabolic contents and citrate synthase activity. Intramyocellular lipid (IMCL) (A), total glucidic (B), glycogen (C) and glucose (D) contents, and citrate synthase activity (E). Data are means ± SEM. *Significantly different from Tp53inp1^+/+^.

No differences were observed between both genotypes for IMCL (Fig. 3A) and total glucidic (Fig. 3B) contents. However, glycogen store was 49% larger (Fig. 3C) in TP53INP1‐deficient mice whereas glucose content was 19% lower (Fig. 3D). Further, the citrate synthase activity did not differ between both groups (Fig. 3E).

### Protein content and oxidation

**Figure 4:**
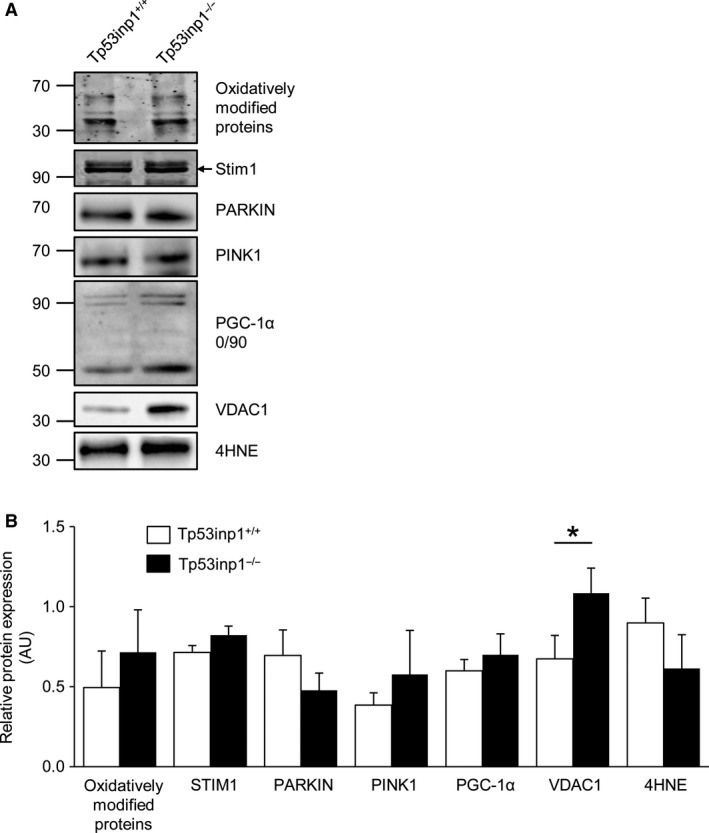
Protein content and oxidation. Gastrocnemius muscle was analyzed by immunoblotting (A) for oxidatively modified protein, STIM1, PARKIN, PINK1, PGC‐1*α*, VDAC1, and 4HNE (B). Data are means ± SEM. *Significantly different from Tp53inp1^+/+^.

There were no differences between both groups for oxidatively modified protein, STIM1, PARKIN, PINK1, PGC‐1*α*, and 4HNE contents (Fig. 4 and B). By contrast, the VDAC1 protein level was 59% higher in TP53INP1‐deficient mice (Fig. 4B).

### Mechanical performance

**Figure 5:**
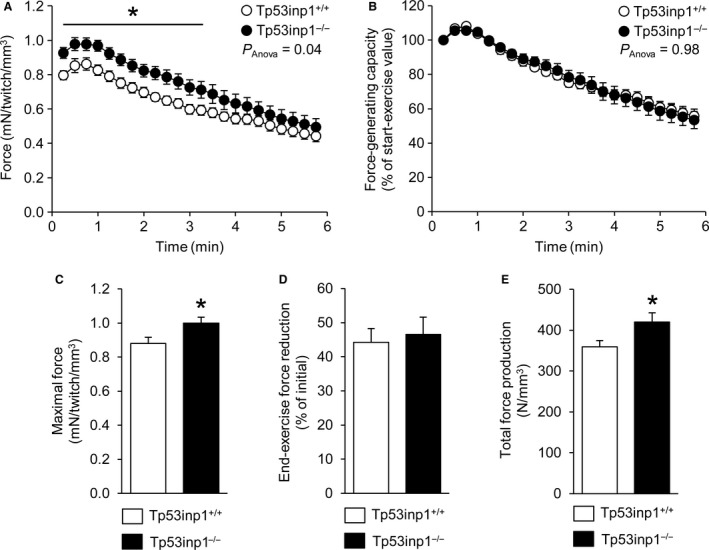
Gastrocnemius muscle mechanical performance. Time courses of force production (A) and force‐generating capacity (B) were measured in vivo throughout the 6‐min fatiguing bout of exercise performed simultaneously to the dynamic ^31^P‐MRS acquisition. Maximal contractile force produced during the whole exercise (C), extent of force reduction measured at the exercise end (D) and total amount of force production during the whole exercise (E). For the panels A and B, *P*_Anova_ indicates the overall result of the two‐way repeated measures analysis of variance, and Tukey post‐hoc multiple comparisons were used to determine pairwise time‐points differences. Data are means ± SEM. *Significantly different from Tp53inp1^+/+^.

Contractile force production was normalized to gastrocnemius muscle volume measured from anatomic hind limb MR images. The time courses of force production and force‐generating capacity throughout the 6‐min in vivo fatiguing bout of exercise are shown in Fig. 5A and B. For each group, force transiently increased in the early stage of the exercise (Fig. 5A) to reach a maximal value that was 13% larger in TP53INP1‐deficient mice (Fig. 5C). Force then decreased progressively until the exercise end as a sign of fatigue development; at this stage, the extent of force reduction did not differ between both groups, averaging around 45% (Fig. 5D). Importantly, force production was 10–15% larger in TP53INP1‐deficient mice during the first half of the exercise (Fig. 5A), whereas the shape of force‐generating capacity curve was very similar for both mouse genotypes during the whole exercise (Fig. 5B). Moreover, the total amount of force produced during the whole exercise was larger (+17%) in TP53INP1‐deficient mice (Fig. 5E).

### Gastrocnemius muscle bioenergetics

**Figure 6:**
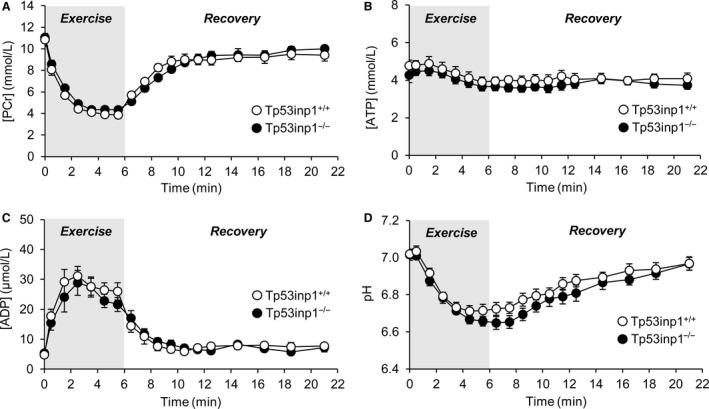
Noninvasive investigation of gastrocnemius muscle bioenergetics using dynamic ^31^P‐MRS. Changes in (phosphocreatine [PCr]) (A), [ATP] (B), [ADP] (C) and pH (D) were measured throughout the 6‐min fatiguing bout of exercise and the 15‐min postexercise recovery period. For each panel, the first time‐point (*t* = 0) indicates the basal value. Data are means ± SEM.

**Figure 7:**
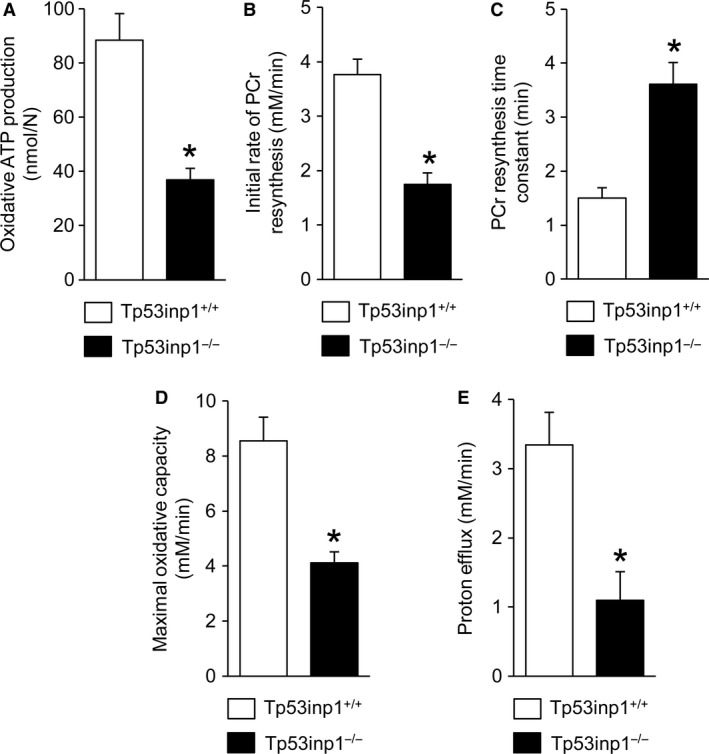
Gastrocnemius muscle oxidative function and proton efflux. Oxidative ATP production at the end of the 6‐min fatiguing bout of exercise (A), initial rate (B) and time constant (C) of phosphocreatine (PCr) resynthesis at the start of the postexercise recovery period, maximal oxidative capacity (D) and proton efflux (E). Data are means ± SEM. *Significantly different from Tp53inp1^+/+^.

Muscle bioenergetics was assessed throughout a standardized rest‐exercise‐recovery protocol. At rest, TP53INP1‐deficient mice did not display any significant alterations of pH and PCr, ATP, and ADP contents (Table 1), which indicates that the basal bioenergetics status was preserved in these animals. During the fatiguing exercise, the time courses of phosphorylated compound levels and pH were comparable in the two mouse genotypes (Fig. 6A–D). At the start of the exercise, PCr was rapidly consumed at a similar rate between wild‐type and TP53INP1‐deficient mice (Table 1). After 2 min, PCr level reached a plateau that was maintained until the end of exercise (Fig. 6A), which indicates the establishment of a metabolic steady state since the PCr pool is at this stage considered as a shuttle for the transport of high‐energy phosphates between the sites of production and consumption of ATP (Meyer et al. 1984; Wallimann et al. 1992). For both groups, ATP level decreased slightly throughout the exercise (Fig. 6B), hence demonstrating that ATP homeostasis was ensured, whereas ADP – the main stimulator of oxidative ATP synthesis in vivo (Gyulai et al. 1985; Kemp and Radda 1994) – continuously accumulated (Fig. 6C) to reach an end‐exercise value that did not differ between mouse genotypes (Table 1). The time course of pH was characterized by a strong and rapid acidosis during the first half of the exercise period followed by a phase of fairly steady state (Fig. 6D). At the exercise end, there were no differences between both genotypes for the extents of PCr degradation (an index of the intramuscular energetics load), ATP depletion and acidosis (Table 1), hence showing that the stress induced by exercise produced similar bioenergetics changes in both groups. Nevertheless, the oxidative ATP production measured at the exercise end was strongly lower (−58%) in mice lacking TP53INP1 (Fig. 7A). During the postexercise recovery period, TP53INP1 deficiency dramatically reduced the initial rate of PCr resynthesis (−54%; Fig. 7B) and prolonged the time constant for PCr resynthesis (+141%; Fig. 7C), which indicates mitochondrial bioenergetics defect. Consistently, the maximal oxidative capacity was reduced in TP53INP1‐deficient mice (−52%; Fig. 7D). In addition, the initial rate of proton efflux from the muscle in the early stage of the recovery was reduced in mice lacking TP53INP1 (−67%; Fig. 7E).

**Table 1:** Gastrocnemius muscle bioenergetics assessed in vivo using ^31^P‐MR spectroscopy

	Tp53inp1^+/+^	Tp53inp1^−/−^
Basal		
[PCr], mM	10.8 ± 0.6	11.1 ± 0.3
[ATP], mM	4.3 ± 0.4	4.8 ± 0.3
[ADP], μM	4.8 ± 0.3	5.4 ± 0.4
pH	7.02 ± 0.03	7.02 ± 0.02
Onset of the exercise		
Initial rate of PCr consumption, mM/min	7.1 ± 0.9	6.7 ± 1.1
End of the exercise		
Δ[PCr] (relative to basal), mM	7.0 ± 0.5	6.7 ± 0.3
Δ[ATP] (relative to basal), mM	0.6 ± 0.3	0.9 ± 0.3
[ADP], μM	26 ± 3	22 ± 3
ΔpH (relative to basal), pH unit	0.33 ± 0.03	0.36 ± 0.04

Data are means ± SEM.

PCr, phosphocreatine.

## Discussion

The main findings are that TP53INP1 deficiency increases whole‐body fat accumulation and adiposity but does not affect oxidative stress, lipid content, and mitochondrial pool in myocyte. However, oxidative ATP synthesis capacity and proton efflux are dramatically reduced in exercising TP53INP1‐deficient muscle in vivo, whereas the fatigue resistance remains unchanged and the contractile force paradoxically increases.

Our data show that TP53INP1 deficiency changes animal morphology. We found that the ratio between body weight and gastrocnemius muscle volume was larger in TP53INP1‐deficient mice, which leads to assumption that body composition is altered in these animals. We have performed in vivo whole‐body MR imaging to test this assumption and found that body and abdominal fat contents were larger (+56% and +50%, respectively) in mouse lacking TP53INP1, which is in line with previous experiments showing that dissected gonadal fat is more abundant in this mouse model (Seillier et al. 2015). Noteworthy, this increased fat content occurred without any concomitant change in lean mass, thereby causing an increased adiposity in mice lacking TP53INP1. Considering that increased adiposity favors the development of metabolic syndrome (Tchernof and Despres 2013; Bhatti et al. 2017), our results therefore support the view that TP53INP1 prevents metabolic syndrome development through the dampening of body fat accumulation (Seillier et al. 2015). More specifically, it has been proposed on the basis of experiments in cultured immortalized mouse embryonic fibroblasts (MEFs) that this propensity to develop metabolic syndrome results from the accumulation of defective mitochondria leading to mitochondria‐derived ROS increase and massive intracellular lipid accretion in the absence of TP53INP1 (Cano et al. 2009; N'Guessan et al. 2011; Seillier et al. 2015). On the contrary, we found that TP53INP1 deficiency did not affect PINK1/PARKIN mitophagy, expression of PGC‐1*α* mitochondria biogenesis factor and creatine kinase activity, which demonstrates that muscle mitochondrial pool and capacity were not changed. Also, we did not detect any difference between both phenotypes for intramyofiber lipid content and biomarkers of reactive oxygen species. Taken together, these data lead us to state that despite skeletal muscle is considered to play an important role in the development of metabolic syndrome, this tissue would not be involved in the ROS‐drive obesity and insulin development induced by TP53INP1 deficiency.

Importantly, our in vivo ^31^P‐MRS measurements however evidenced that muscle mitochondrial capacity was dramatically impaired in TP53INP1‐deficient mice under physiological condition. This impairment did not affect the basal bioenergetics status but strongly reduced the normalized oxidative ATP synthesis in exercising muscle, that is, when energy demand is high. The origin of this oxidative defect has to be explained. We found that the time constant of PCr resynthesis after the 6‐min fatiguing bout of exercise was increased in muscle lacking TP53INP1, which indicates (Lanza et al. 2011; Prompers et al. 2014) that the intrinsic mitochondrial capacity was reduced in vivo. It must be pointed that mitochondrial respiration is mainly stimulated in vivo by ADP throughout a feedback loop (Gyulai et al. 1985; Kemp and Radda 1994). However, we found that oxidative ATP production was reduced in TP53INP1‐deficient mice whereas ADP accumulation did not differ between both groups. Then, it can be assumed that lack of TP53INP1 weakens the feedback control of ADP on mitochondrial respiration. In that, we found that VDAC1 content was increased in muscle lacking TP53INP1, in accordance with what was observed in cultured TP53INP1‐deficient MEFs (Seillier et al. 2015). VDAC1 has a key role in the regulation of mitochondrial bioenergetics, being involved in the transport of ADP across the mitochondrial outer membrane (Rostovtseva and Colombini 1997; Anflous et al. 2001). Therefore, increased VDAC1 content in TP53INP1‐deficient mice can be interpreted as a compensatory mechanism for alleviating the potent weakened feedback control of ADP. Besides, the ability of mitochondria to produce ATP critically depends on oxygen supply. Yet, we found that proton efflux from the muscle was 67% lower in TP53INP1‐deficient mice, which indicates (Kemp et al. 1995; Cea et al. 2002) reduction in muscle blood flow. Such a reduction is consistent with previous studies showing that oxidative stress impairs the endothelial function via accelerated degradation of nitric oxide (Goodwill and Frisbee 2012; Sena et al. 2013). Blood flow reduction might limit oxygen and substrate delivery to the muscle, which in turn would reduce oxidative ATP synthesis during exercise.

Given the deleterious effect of TP53INP1 deficiency on oxidative capacity, one can wonder whether TP53INP1 deficiency impairs ATP regeneration in exercising muscle, thereby affecting muscle function. ATP is indeed critical for the contractile process because it is involved in myofilaments sliding and ion transport (including calcium reuptake by the sarcoplasmic reticulum). Additionally, skeletal muscle has a high potential for regenerating ATP during exercise to keep in pace with an energy demand that can increase by several orders of magnitude at the transition between rest and exercise (Wallimann et al. 1992; Hochachka and McClelland 1997; Sahlin et al. 1998). In the present study, we have implemented an intensive exercise protocol to produce a global energetic stress. Despite this, we found that ATP level and energy load throughout the exercise were very similar in the two groups, which demonstrate that TP53INP1 deficiency did not compromise ATP homeostasis. We found that muscle fatigue resistance was not affected by TP53INP1 deficiency. On the other hand, we surprisingly observed that both the maximal contractile force and the total amount of force produced during the whole fatiguing exercise were larger (+13% and +17%, respectively) in TP53INP1‐deficient mice. These data could not be linked to any typological change because we found that lack of TP53INP1 did not affect MHC distribution. Also, it must be kept in mind that muscle performance can be improved in exercising muscle in response to an increase in energy supply and/or calcium release from the sarcoplasmic reticulum (Allen et al. 1997; Westerblad et al. 1998). However, we found that gastrocnemius muscle content of STIM1, the main regulator of Store Operated Calcium Entry in the skeletal muscle (Boncompagni et al. 2017), did not differ between TP53INP1‐deficient and wild‐type mice, thereby suggesting (Bohm et al. 2014) that lack of TP53INP1 did not affect intracellular calcium homeostasis. Further investigations are required to determinate the origin of the ergogenic effect associated to TP53INP1 deficiency.

In conclusion, this study provides data evidencing that TP53INP1 plays a role in muscle function and bioenergetics. TP53INP1 deficiency actually reduces the in vivo oxidative capacity in exercising muscle possibly as the consequence of abnormal mitochondrial respiration regulation and/or defective blood supply. Nevertheless, TP53INP1 deficiency does not affect oxidative stress level and mitochondrial pool in resting muscle, thereby indicating that this tissue is not involved in the development of metabolic syndrome previously reported in TP53INP1‐deficient mice.

## Conflict of Interest

The authors have no conflicts of interest, financial or otherwise, to declare.
